# Improving the RNA velocity approach with single-cell RNA lifecycle (nascent, mature and degrading RNAs) sequencing technologies

**DOI:** 10.1093/nar/gkad969

**Published:** 2023-11-06

**Authors:** Chen Zhang, Yitong Fang, Weitian Chen, Zhichao Chen, Ying Zhang, Yeming Xie, Wenfang Chen, Zhe Xie, Mei Guo, Juan Wang, Chen Tan, Hongqi Wang, Chong Tang

**Affiliations:** BGI, Shenzhen 518000, China; BGI, Shenzhen 518000, China; BGI, Shenzhen 518000, China; BGI Education Center, University of Chinese Academy of Sciences, Shenzhen 518083, China; BGI, Shenzhen 518000, China; Guangdong Provincial Reproductive Science Institute (Guangdong Provincial Fertility Hospital), Guangzhou, China; NHC Key Laboratory of Male Reproduction and Genetics, Guangzhou, China; BGI, Shenzhen 518000, China; BGI, Shenzhen 518000, China; BGI, Shenzhen 518000, China; BGI, Shenzhen 518000, China; BGI, Shenzhen 518000, China; BGI, Shenzhen 518000, China; BGI, Shenzhen 518000, China; BGI, Shenzhen 518000, China

## Abstract

We presented an experimental method called FLOUR-seq, which combines BD Rhapsody and nanopore sequencing to detect the RNA lifecycle (including nascent, mature, and degrading RNAs) in cells. Additionally, we updated our HIT-scISOseq V2 to discover a more accurate RNA lifecycle using 10x Chromium and Pacbio sequencing. Most importantly, to explore how single-cell full-length RNA sequencing technologies could help improve the RNA velocity approach, we introduced a new algorithm called ‘Region Velocity’ to more accurately configure cellular RNA velocity. We applied this algorithm to study spermiogenesis and compared the performance of FLOUR-seq with Pacbio-based HIT-scISOseq V2. Our findings demonstrated that ‘Region Velocity’ is more suitable for analyzing single-cell full-length RNA data than traditional RNA velocity approaches. These novel methods could be useful for researchers looking to discover full-length RNAs in single cells and comprehensively monitor RNA lifecycle in cells.

## Graphical Abstract

**Graphical Abstract:**
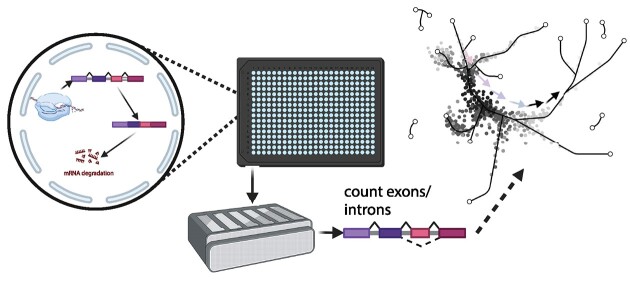

## Introduction

Single-cell sequencing has been a significant area of study for many years. There are various methods for single-cell sequencing, with the most popular being single-cell RNA sequencing (scRNA-seq), which measures RNA expression in a single cell. Single-cell transcriptome analysis is a powerful approach to mapping genotypes to phenotypes, which has been a long-standing challenge in biology (1). The scRNA-seq addresses the limited volume of samples, uncovers complex and rare cell populations, regulatory relationships between genes and trajectories of distinct cell lineages in development (1). However, recent researches have shown that the measurement of gene expression alone is insufficient to meet the needs of further studies. Scientists have turned their attention to the dynamic structure of transcriptomes, such as transcription start/end sites, exons, splice sites and RNA editing sites, to more precisely define genotypes (2). Alternative splicing, which is a form of RNA dynamic structure, greatly expands the coding capacities of finite genomes, contributing to functional proteomic complexity (3), human disease (4), the functionality of the nervous system (5) and cell differentiation (6). Therefore, the technology of full-length scRNA-seq, which fully discloses the RNA dynamic structure in single cells, could provide valuable new insights into complex biological systems.

Most conventional scRNA-seq technologies rely on the Smart-seq concept (7–11). The Moloney Murine Leukemia Virus (MMLV) reverse transcriptase is used to reverse transcript the single-cell RNAs to cDNAs and tag adaptors to the 5′ ends via template-switching activity. The cDNAs are then amplified by PCR and fragmented by transposon (Tn5) for next-generation sequencing (NGS). The Tn5-enabled and unique molecular identifier-guided (UMI) amplicon assembly can bioinformatically assemble the full-length RNA (12,13). However, due to fragmentation, these methods have not provided a reliable picture of complete RNA isoforms (2). Third-generation sequencing overcomes this limitation and allows for end-to-end sequencing of entire RNA/cDNA molecules. The Pacbio sequel (fluorescent-based) and Nanopore (electro-osmotic flow-based) are two popular third-generation sequencing technologies that benefit non-model organisms (14), novel transcript discovery (15), and complex isoform dynamics in immunology (16,17). However, sequencing accuracy and throughput remain major challenges for scientists working in single-cell third-generation sequencing. Single-cell sequencing requires high sequencing accuracy to effectively categorize transcriptomes based on similar cell barcodes and high-throughput ability to produce >200 million reads for thousands of cells. To address the need for precisely sequencing cell barcodes, single-cell isoform RNA-seq (SciSOr-seq) leverages the Pacbio accuracy (99.2%) to partially sequence the single-cell library and combine NGS for analysis (18). However, Pacbio generates 100x fewer reads than NGS, and additional full-length data is required to address sequencing gaps. Our group developed HIT-scISOseq, which ligates multiple reads into a single molecule, quadrupling the throughput on the Pacbio platform (19). The throughput limitation of Pacbio prompted scientists to use nanopore sequencing with higher throughput. However, the low sequencing accuracy of nanopore makes it difficult to effectively demultiplex similar cell barcodes. Single-cell full-length transcript by sampling (FLT-seq) uses the FLAME pipeline to directly filter and demultiplex cell barcodes (20). K. Lebrigand introduced an approach that combines nanopore sequencing with UMI to obtain error-corrected cell barcodes with NGS assistance (21). To further improve nanopore accuracy, several groups used Rolling Circle to Concatemeric Consensus (R2C2) to sequence the same sequence multiple times (16,22–24). With R2C2 technology, accuracy increases but productivity decreases due to concatemeric reads. An alternative method is optimized cell barcode sequence distance. Single-cell corrected long-read sequencing (scCOLOR-seq) synthesizes optimized cell barcodes using homodimeric nucleoside phosphoramidite building blocks, providing a means for sequencing-error detection and correction of barcodes and UMIs (25). The advantage of this method is somewhat lessened due to the difficulty of bead-based barcode synthesis. Additionally, most full-length single-cell technology is biased towards long fragments (>500 bp) and ignores degrading short RNAs, which may affect cell status assessment. This motivates the need for an alternative approach that is more accessible for researchers and comprehensively monitors RNA lifecycle (nascent, mature, degrading RNAs) in cells.

The rapid growth of the full-length scRNA-seq field has created a high demand for computational analysis of full-length RNA single-cell data. However, most software has focused on the challenging debarcoding process (20). Only a limited amount of software has been developed specifically for functional analysis of full-length scRNA-seq data for biologists, such as cell subpopulation identification, cell lineage, and pseudotime reconstruction, etc. (26). While most short-read scRNAseq software could be adapted for full-length scRNAseq data, it often compromises full-length RNA information. Previously, we have used full-length scRNA-seq for transcript-level cell subpopulation identification (20,27,19), and to detect cell-specific RNA splicing patterns (28). The recently developed concept of ‘RNA velocity’ deals with dynamic changes in mRNA expression and complements scRNA-seq data to predict cell lineage and pseudotime reconstruction by quantifying the time-dependent relationship between the abundance of precursor (unspliced transcripts, u) and mature mRNA (spliced transcripts, s) (29,30). However, there is currently limited ‘RNA velocity’ software designed for full-length scRNA-seq. Given the sensitivity of spliced RNA detection using full-length scRNA-seq, we are interested in whether it could potentially outperform short-read scRNA-seq after optimization.

Taking these needs into consideration, we presented three barcoded technologies (BD Rhapsody) that are accessible to most researchers: high-throughput single-cell ONT full-length RNA sequencing (FLOUR-seq). The ultralong three barcodes can be discriminated using nanopore technology with 70% debarcoding efficiency and can detect the RNA lifecycle (nascent, mature, degraded RNAs). We also updated our HIT-scISOseq V2 to detect the RNA lifecycle. We used these technologies to study spermiogenesis, which not only sequenced the full-length nascent RNA, but also sequenced the degrading RNAs, reflecting the RNA velocity of the cells. Moreover, we developed the novel algorithm ‘Region velocity’ specifically for full-length scRNA-seq to configure cellular RNA velocity more accurately. The FLOUR-seq/HIT-scISOseq V2 and region velocity algorithm can enhance full-length scRNA-seq growth in the near future.

## Materials and methods

### Cell culture

Derivative human cell line which expresses a mutant version of the SV40 large T antigen (HEK 293T) (abclonal) and mouse 4T1cell line (abclonal) were each maintained in DMEM-high glucose (Thermo Fisher 11995065) supplemented with 10% fetal bovine serum (FBS) (Thermo fisher 1009141).

### Preparation of spermiogenesis cells

All mice used in this study were on the C57BL/6J background, and housed under specific pathogen-free conditions in a temperature- and humidity-controlled animal facility at the Nantong University. The animal operation protocol used in this study was approved by the Animal Care and Use Committee of Nantong University. Eight testes were pooled each time for cell preparation. After being removed and decapsulated, testes were placed into 10ml of the EKRB buffer containing 5mg collagenase (Sigma) for a 12-min digestion at 32°C to disperse the testicular cells. Once dispersed, the testicular cells were washed three times using the EKRB buffer followed by trypsin digestion by incubation in 10ml EKR buffer containing trypsin (Sigma, 0.25 mg/ml) and DNase I (Sigma, 20 μg/ml) at 37°C for 12min with occasional pipetting to facilitate cell dispersion. Fully dispersed testicular cells were washed three times followed by centrifugation and re-suspension in 10 ml of 0.5% BSA. The cell suspension was passed through 50-μm filters and the filtrate was saved as single cell suspension.

### Experiment procedure of FLOUR-seq

- single cell processing with BD Express Platform

The dissociated single cells were processed using the BD ExpressTM Platform, following some of the manufacturer's recommendations. This included performing a Single cell 3′ whole transcriptome amplification (WTA) with sample multiplexing kit (SMK) and AbSeq protocol, using the BD RhapsodyTM Cartridge Reagent Kit (633731, BD biosciences), BD RhapsodyTM Cartridge Kit (633733, BD biosciences), and BD RhapsodyTM cDNA Kit (633773, BD biosciences).

To elaborate, 20 000 dissociated cells and Cell Capture Beads were loaded into a pre-treated BD Rhapsody Cartridge, followed by lysing the cells with lysis buffer. The beads were washed with Bead Wash Buffer, and reverse transcription was performed. The tube containing the beads was placed on a magnet, and the 200 μl of suspension was moved to a new tube and saved for the following template switching reaction. Finally, the beads were treated with exonuclease I.

- Template switching of cDNA and PCR amplification

The beads treated with exonuclease I were resuspended in the saved 200 μl reverse transcription reaction mix, supplemented with 5 μl of 100 μM template switching oligo (AAGCAGTGGTATCAACGCAGAGTACrGrG + G) and 2 μl of 1 M MgCl_2_. They were then incubated on the thermomixer for 30 min at 1200 rpm and 42°C. The tube was placed on a magnet and the supernatant was removed. The beads containing cDNA were suspended in 200 μl of PCR reaction mix, comprised of 100 μl of KAPA HiFi HotStart 2x ReadyMix (Kapa Biosystems), 10 μl of PCR primer 1 (5′-GCTGTACCGATGCTAGGCTGTTCGTCACCATAGTTGCGTCTCATG-3′), 10 μl of PCR primer 2 (5′-AATGATACGGCGACCACCGAGATCTACACTATAGCCTACACTCTTTCCCTACACGACGCTCTTCCGATCT-3′), and 80 μl of nuclease-free water. The beads were fully resuspended, and 25 μl of PCR reaction mix with beads was pipetted into each of eight 0.2-ml PCR tubes. The PCR reaction was performed in a PCR thermal cycler for 12 cycles, followed by purification with 0.8x Agencourt AMPure XP Beads (Beckman Coulter).

The amplified PCR products underwent end-repair, A-tailing, and ligation with a sequencing adapter using the Ligation Sequencing Kit (SQK-LSK110, Oxford Nanopore Technologies). The library was then sequenced on the Oxford Nanopore PromethION sequencing platform.

### Experimental procedure of HIT-scISOseq V2

The dissociated single cells were processed through the GemCode Single Cell Platform per manufacturer's recommendations using the Chromium Single Cell 3′ GEM, Library, and Gel Bead Kit v3 (10x Genomics; PN-1000075). In V2 version, we used exonuclease I to treat the solution after emulsification. In the template switching, we used the different template switching primer AAGCAGTGGTATCAACGCAGAGTACrGrG + G. cDNA products were amplified with KAPA HiFi HotStart Uracil 2 x ReadyMix (Kapa Biosystems) and newly designed PCR primers that contain a deoxyuraciland one (5 different primer sets for 5 PCR reactions, in order to create 1–2-3–4-5 sequential ligations). The pooled PCR products from 5 PCR reactions were digested by USER Enzyme (NEB) and ligated together by T4 DNA ligase and processed to the Pacbio sequencing.

### SLAM-seq

- Cell preparation and metabolic labeling

Hek293T (ATCC, CRL-3216) cells were grown in 10 cm tissue culture plates to approximately 50% in DMEM media supplemented with 10% FBS and 1% P/S. Four plates of cells were treated with 4-thiouridine at 200 μM final concentration at 37°C for 2, 4, 6, 8 h respectively. Cells were trypsinized, pelleted by centrifugation at 300g for 5 min and washed once with ice-cold PBS. Cells were then proceed immediately to modified RNeasy isolation.

- Modified RNeasy isolation

Total RNA of Cells was extracted with RNeasy Mini Kit (74104, QIAGEN) according to manufacturer's recommendations with tiny modification. Briefly, cells were pelleted by centrifugation, resuspended in 350 μl RLT buffer supplemented with 35 μl BME (1% final). 350 μl freshly prepared 70% ethanol was added to the lysis mixture and mix well by inversion and the mixture was passed through an RNeasy spin column. The column was washed with buffer RW1, buffer RPE supplemented with 50 μl BME (1% final), and freshly prepared 80% ethanol. RNA was eluted for the column with 15 μl nucleases-free water.

- Chemistry treated of RNA

2 μg of isolated total RNA was added to a mixture of TFEA (600 mM), EDTA (1 mM) and sodium acetate (pH 5.2, 100 mM) in water. A solution of NaIO4 (10 mM) was then added to the tube and flicking mixed, and the reaction mixture was incubated at 45°C for 1 h. Total RNA was purified with 1.8x volumes RNAClean XP beads. 18 μl of RNA was added to 2μl of a 10× reducing master mix contain 100 mM Tris–HCl, pH 7.4, 100 mM DTT, 1 M NaCl and 10 mM EDTA, pH 8, and incubate at 37°C for 30 min. Total RNA was purified with 1.8× volumes RNAClean XP beads and assessed concentration and quality of RNA by bioanalyzer.

- Poly A length Iso-seq library construction

Poly A length Iso-seq library was constructed as previous description (31). Briefly, 500 ng of RNA was added to an extension mix of 1× NEBuffer 2, 0.2 U RNase Inhibitor (Enzymatics), 0.5 mM each dNTP, 0.5 uM PA-RT-primer (5′-GTCTCGTGGGCTCGG AGATGTGTATAAGAGACAG NNNNNN dUTTTTTTTTTTTTTTTTT /3Inverted dT/) and 0.5 U DNA Polymerase I, Large (Klenow) Fragment and incubated at 37°C for1h. 40mM EDTA was added and incubated at 37 C for 20 min to stop reaction, and 1 μl of USER enzymes (NEB) were add to the mixture and incubated at 37°C for 30 min. The samples were purified with 1.8× RNAClean XP Beads. The end-extended RNA was reverse transcribed with template switching similarly to Smart-seq2 method with RT primer and TSO. The cDNA was purified with 0.8× AMPure XP beads. PCR amplification was performed by using KAPA HiFi HotStart ReadyMix (KAPA Biosystems) with PCR primers foolowed by 0.8× AMPure XP beads.

- Nanopore sequencing

Amplified PCR products were end-repaired, A-tailed, and ligated with a sequencing adapter using Ligation Sequencing Kit (SQK-LSK110, Oxford Nanopore Technologies). The library was sequenced in Oxford Nanopore promethION sequencing platform.

### Demultiplexing barcodes

- Reference construction

BD cell label is roughly composed of five sections, including three separated barcodes of 9bp and two known linkers. Each barcode is randomly selected from a pool of 96 pre-defined sequences which allowed us labeling up to 96^3^ cells. The minimum Hamming distance of three barcodes was 4 bp and the mean distance was 6.6 bp. If taking insertion, deletion and substitution events into account, their minimum Levenshtein distance was 2 bp and the mean was 5.7 bp. In comparison to 10X barcode, BD barcode takes the advantage of its longer design and therefore has a greater edit distance.

We constructed the cell label reference by generating 96^3^ combinations of 9bp barcodes joined by two known linker sequences, so at this stage, the reference should begin with the first barcode and end with the third barcode. However, problems would arise with this type of reference because its flanking regions may be clipped off during alignment. In order to avoid this issue, we elongated the reference with more sequences to act as the anchor, including binding site, UMI and oligo-dTs. Therefore, the final version of reference had a length of 98 bases, and we added more Ns to both ends to ensure the reference is longer than reads. For details, please refer to our algorithm AASRA (32).

- First round of demultiplexing

We took a subset of sequencing data and had a rough inspection of the library. Based on the positions of oligo dT, around 47% of the reads were classified as forward strands and 40% reads were reverse strands. Nearly 60% reads could be fully aligned to 5′ and 3′ binding site, indicating most of the reads possessed a complete library structure.

In order to find the most appropriate method to assign barcodes, we examined the performance of various aligners including minimap2, bwa-mem, bowtie2 and BLASTN. Although minimap2 was usually recommended for long read alignment, only <25% reads could be successfully demultiplexed. Similarly, many reads with the correct cell label were either wrongly assigned or unmapped in bwa-mem algorithm. As such, they were less recommended to use in this case where the length of query sequence was much longer than the reference. In comparison, the local alignment mode of bowtie2 and BLASTN outperformed other tools by having a mapping rate of 57.87% and 76.92%, respectively. BLASTN alignment generated less gaps, mismatches and had a longer mean aligned length than bowtie2, suggesting a better accuracy. However, bowtie2 was superior due to its ultrafast and memory-saving features. The speed of demultiplexing was further boosted when the read was trimmed into 300 bp based on theoretical positions of barcodes. For example, bowtie2 and BLASTN could demultiplex 400 000 and 4000 reads respectively under the same clock time. All these results suggested that bowtie2 was much more efficient than BLASTN in this case, thus the local alignment mode of bowtie2 became the optimal choice for the first round of demultiplexing.

- Validation and optimization of the first round of demultiplexing

**Figure 1.**
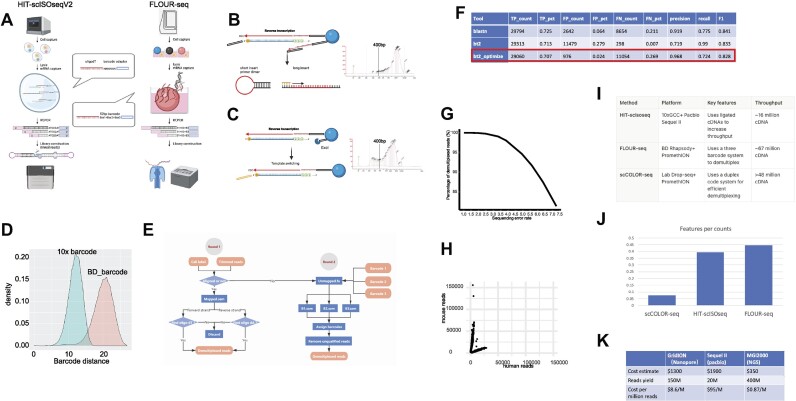
The FLOUR-seq experimental and bioinformatic procedure. (**A**) In a previous study, we developed the HIT-scisoseq platform for full-length single-cell sequencing, using the 10× Genomics Chromium Controller (10× GCC) and Pacbio Sequel II. Cells were captured in droplets and labeled by 1 million Gel Bead-In EMulsions (GEMs) with a 9bp barcode sequence. Reverse transcription then tagged the other adaptor on the 5′ end by the template switching activity. PCR amplification incorporated specific uracil-containing primers. USER-generated sticky end PCR fragments were linked together as head-to-tail ligation, producing 5–4 fold (16 million) more reads than conventional Pacbio Sequel II (4 million). For more details, please refer to our previous study. In this study, we aimed to generate 100 million cDNA reads at a low cost, using the high-throughput nanopore sequencing. To achieve this, we developed the HISOFA-seq platform, which combines the BD Rhapsody system and nanopore sequencing. Single cells were captured in microarray and labeled with beads having 52 bp mega barcodes, with a large Levenshtein distance among them to compensate for the accuracy deficiency (70%∼90% accuracy) of nanopore sequencing. (**B**) In the reverse transcription below, we utilized the same sequence tagging method from our previous HIT-scISOseq study (Zheng, 2020), as well as other studies. This method inhibited amplification of dimmer/short fragments (<500 bp) with the stem-loop structure at the 5′ end. The Agilent2100 was used to display the distribution of the amplified cDNA. However, it should be noted that many fragments smaller than 500bp were not amplified. (**C**) To sequence full-scale RNAs (nascent, stable, short degrading RNAs) and avoid dimmer amplification, we used exonuclease I (ExoI) to remove excess primers on beads after reverse transcription and then tagged the other 5′ adaptor on recycled beads. PCR efficiently amplified full-scale cDNAs, offering a holographic view of the natural transcriptomic landscape. The Agilent2100 was used to display the distribution of the amplified cDNA. It should be noted that the fragments smaller than 500 were amplified. (**D**) The density plot of BD barcode and 10X barcode edit distances shows that BD barcodes (HSOFA-seq) have 2x larger distance than the 10× barcode system. (**E**) The barcode demultiplexing process was done following a two-step pipeline for BD barcodes sequenced with the Oxford Nanopore platform. The first step was done by aligning reads to the BD cell label whitelist. The result was then validated by the presence of oligo dT in forward aligned reads and oligo dA in reverse aligned reads. Any unaligned reads were collected and aligned to three barcodes separately. Only reads aligned to all three barcodes were regarded as successfully recovered reads. (2nd round is optional for many users). (**F**) The performance of the first round of demultiplexing with different algorithms was analyzed. HISOFA-seq was based on the bt2_optimized algorithm, which has comparable specificity and sensitivity with blastn. (**G**) Simulated data showing the percentage of demultiplexed reads while increasing sequencing error rate. 10,000 reads with a variety of error rates were simulated with ART software followed by our demultiplexing pipeline. The percentage of recovered reads was calculated for different error rates. (**H**) The dot plot of human-mouse collision was created using a mixture of human and mouse cells to estimate barcode collision. (**I**) Comparing full-length single-cell sequencing technologies. (**J**) The feature per count is the mean of the gene features per cell divided by the mean of the read counts per cell. (**K**) Cost comparison among different sequencing platforms.

Our demultiplexing strategy was further validated using simulated data, which was created for the purpose of mimicking the real condition by introducing sequencing errors and PCR errors (33). As Figure 1F shows, the true positive rate (TPR), false positive rate (FPR) and false negative rate (FNR) of BLASTN were 72.5%, 6.4% and 21.1%, respectively. The high precision value (0.919) also indicated the result was reliable enough for downstream analysis. In comparison, bowtie2 had a much worse precision of 0.719 due to its high FPR (27.9%), which would affect the accuracy of our demultiplexing result and subsequent analyses. Therefore, we carried out more attempts on improving and optimizing on bowtie2 parameters in order to reduce its false positive to the minimal level. The optimized alignment had a TPR of 70.7% and its FPR was dramatically reduced to 2.4%, which was even lower than BLASTN. Due to its high TPR and low FPR, bowtie2 with optimized parameters turned out to be the best strategy for the first round of demultiplexing.

- Second round of demultiplexing

This step is optional. During the first round of demultiplexing, the procedure of trimming the reads into 300 bp was strict and harsh, and those reads with barcode outside the proposed positions might be lost. In addition, it could not tolerate the cases when three barcodes were linked by defective linkers. Therefore, it was necessary to rescue those reads with another round of demultiplexing.

In contrast to the previous step where reads were aligned to the cell label, we did not use cell label as the reference for the second step. Three barcodes were individually aligned to the reference, which was constructed by all unassigned reads from the first round of demultiplexing. If a read was aligned to all three barcodes and they were separated by an interval of similar length to the linker sequence, it will be assigned to the corresponding cell label. Using the test dataset, this procedure could further rescue 13.7% reads, which increased the demultiplexing rate from 57.87% into 71.58%. All unmapped reads will be discarded for downstream analysis.

### Single-cell short read analysis

Single-cell expression matrix was generated by standard 10× Genomics CellRanger pipeline (version 3.1.0) (34).

### Pacbio single-cell isoform sequencing analysis

Generation of Circular Consensus Sequencing Reads, generation of Single Cell Full-Length Non-Concatemer (FLNC) Reads, genome alignment of FLNC reads, Cell Barcode correction and UMI correction, collapsing redundant isoforms, were performed according to our previous HIT-scISOseq (19).

### Nanopore Single-cell isoform sequencing analysis

- Genome alignment

The Full length of reads from the previous steps are mapped to a supplied reference genome (GCF_000364345.1_Macaca_fascicularis_5.0, mm10 (GENCODE vM23/Ensembl 98)) using minimap2 (v2.17-r954-dirty) (35) with the following parameters: -ax splice -uf –secondary = no -C5. The samtools (v1.9) (36) was used to compress sam files into bam, sort bam files and index reference files.

- Generate gene-barcode matrix

spliced_bam2gff (https://github.com/nanoporetech/spliced_bam2gff) with default parameter was used to convert spliced bam alignments into GFF2 format, The GFF2 file was then compared with the reference comments using the gffCompare(v0.11.6) (37) tool. Besides the exon related class code :‘ = c k m n j e o ’, intron related class_code ‘i,y’ was selected for further analysis. Then gene-barcode matrix was generated by Barcode file, UMI file read and gene mapping file through our custom script.

- Collapsing redundant isoforms

We used cDNA_Cupcake python scripts (https://github.com/Magdoll/cDNA_Cupcake) python script collapse_isoforms_by_sam.py to collapse redundant isoforms, the parameters were set: as -c 0.95 -i 0.95 –max_fuzzy_junction 5 –max_5_diff 1000 –max_3_diff 30.

- Generate exon and intron matrices

In order to know whether reads are from spliced transcripts or unspliced transcripts, we need to see if reads contain intronic sequences. For short reads RNA single cell sequencing, exon and intron count matrices were peformed by velocyto (v0.6) (38). For long reads RNA single cell sequencing, the known genomic exon and intron coordinates were extracted from the GTF annotation file, and the overlapping coordinates of exon and intron coordinates were merged using BedTools merge respectively. Then we extracted the coordinates of splice alignment of observation data and compared them with known exon and intron annotation through BedTools intersect. Some researchers believe that the intron length is greater than 50bp (39), while others believe that the minimum intron length is 20 bp (40). Therefore, an intersection with known intron with a length >20 bp was considered as an intron, where the count of intron with the length of 20–50 bp accounts for only 0.5% of the known genomic intron annotation file.

- Generate spliced and unspliced matrices

A long read with intronic sequences were considered as unspliced transcripts and cell-gene-unspliced/unspliced matrices was generated by our custom python script.

- Cell-calling using gene-barcode matrix

R package Matrix (v1.4) was used to load data as sparse Matrix, and barcodeRanks from DropletUtils(v1.2.2) (41) was used to calculate the Inflection and Knee of barcode rank and UMI distribution plot. And then the identification of cells from empty droplets was performed by emptyDrops function (41), threshold of gene counts (less than 20) for barcodes were specified as background. FDR (0.01) was used for testing whether a barcode is an empty cell.

Cells calling according to the number of UMIs associated with each barcode performed by defaultDrops function (41). Finally, Estimated Number of Cells, Total Genes Detected, Mean Genes per Cell, Median Genes per Cell, Mean UMI Counts per Cell, Median UMI Counts per Cell, Mean Reads per Cell, Median Reads per Cell and Fraction Reads in Cells, as implemented in CellRanger, were calculated by customed R script. Additionally, the h5 could be reanalyzed by Cellranger by handing the output of droputils.

### Processing scRNA-seq data for velocity analysis

Expression matrices generated above were imported to Seurat 4.1.0 (42) or MUDAN 0.1.0 (43), which were first log normalized and scaled. The number of principal component analysis (PCA) was mainly determined by the elbow graph, which guided the unsupervised clustering. The number of clusters were determined by multi-resolution through clustree 0.4.4 (44). The clustering results were mainly displayed and analyzed by tSNE and UMAP. Cell populations were mainly determined by marker genes.

### Theory of region velocity

**Figure 2.**
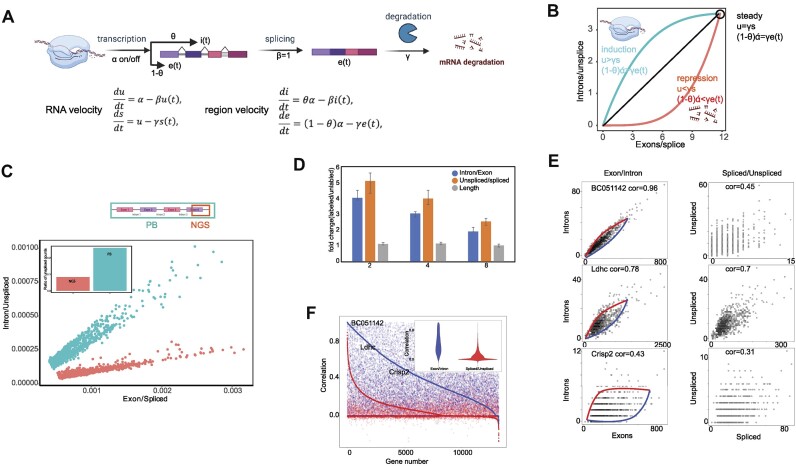
Principles and performance of region velocity. (**A**) This model of RNA transcription dynamics captures the ratio of introns (θ), transcription (α), splicing (β), and degradation (γ) rates. It shows the change of exons and introns. (**B**) To simulate exons and introns, we used region velocity. The simulation predicts the time of exons and introns from 0 to four times the transcription change time (ts) using parameters from Region velocity. An analytical formula is used to determine the exons and introns, and each point represents a cell. Blue indicates cells in the transcription process, while red indicates cells in the splicing or degradation process. The black circle indicated the steady state. The parameters u and s in the formula indicate the steady state of the conventional RNA velocity. The i(t) and e(t) refer to our new region velocity model. (**C**) The difference of spliced counts and unspliced counts between NGS and Pacbio data, including their distribution and ratio, is indicated by red and blue, respectively. The histogram shows the ratio of unsplice/introns in NGS and Pacbio data. (**D**) The figure displays changes in intron/exon, unsplice/splice, length in both the 4sU-labeled and unlabeled RNAs. The x-axis represents the time of 4sU labeling, while the y-axis shows the fold changes between labeled nascent RNAs and unlabelled mature RNAs. Data points are color-coded based on parameters such as unsplice/spliced, intron/exon, and length. (**F**) The accompanying figure displays the correlation between exons and introns, as well as between spliced counts and unspliced counts. The lines on the graph are arranged based on the correlation coefficient, from highest to lowest. Blue lines indicate the correlation between exons and introns, while red lines indicate the correlation between spliced counts and unspliced counts. (**E**) The three genes on the left represent three quantiles of their correlation values between exons and introns. On the right, the corresponding genes of unspliced counts and spliced counts are chosen to further demonstrate the difference.

The model is shown in Figure 2A. The theory of Region velocity is inspired and inferred by RNA velocity. Region velocity includes steady-state model and dynamical model using EM algorithm. The detailed inference and computational framework are elaborated in supplementary notes. Simulation of exons and introns using Region velocity is completed by computation framework of EM algorithm excluded iteration step (step 4 in supplementary notes) to solve the switch time from transcription process to splicing and degradation process. With switch time, equation 11 and 12 in supplemental notes using parameters from steady-state model are used to simulate the expected exons and introns counts.

### Velocity analysis pipeline

RNA velocity is implemented in R package (velocyto.R) (38) of original framework. All greedy balanced KNN algorithm in two samples used the default parameters. The velocity is estimated using gene.relative.velocity.estimates function with parameters ‘fit.quantile = 0.05, min.nmat.emat.correlation = 0.2, min.nmat.emat.slope = 0.2, kCells = 10’ from expression matrices of unspliced and spliced counts. The projection plot is drawn using show.velocity.on.embedding.cor function with parameters ‘show.grid.flow = TRUE’. Region velocity is implemented in new R package – Regionvelocity (https://github.com/Dekayzc/Regionvelocity) which contained steady state model and dynamics model from matrices of exons and introns counts.

### Region velocity pipeline of VASA-seq

Raw data of Mouse ES cells were downloaded from NCBI with SRA number SRR14783103 and SRR14783084 for E8.5 and E9.5 respectively. The cell barcode file was first generated from first sixteen bases of reads 1. Then, final cell barcodes were defined by barcodeRanks from DropletUtils(v1.2.2) (41). With barcode files and raw fastq files, we used pipeline of VASA-seq to obtain bam result (45). From bam result, we used custom script to generate exon and intron matrixes with software assistance of spliced_bam2gff, bedTools and gffCompare, of which the parameters could be referred to analysis of FLOUR-seq and HIT-scISOseq above. The gene counts were defined by unique fragment index (UFI) of VASA-seq. Also, exon and intron counts of each gene were also determined by UFI. The same cell barcodes in both E8.5 and E9.5 group were removed. With exon and intron matrixes, we used region velocity package to run velocity analysis of VASA-seq which contained mixed data of E8.5 and E9.5. The cell annotation was determined by two cell barcode files which included E8.5 and E9.5 respectively. The projection plot was drawn using show.velocity.on.embedding.cor function with parameters ‘show.grid.flow = TRUE’.

## Results

### Principle and development of FLOUR-seq

In our previous research (19), we developed the HIT-scISOseq platform, which provides full-length single-cell sequencing with 20 million reads. This platform used the 10x Genomics Chromium Controller (10xGCC) and Pacbio sequel II (Figure 1A) to capture cells in droplets and label them with 1 million GEMs (Gel Bead-In EMulsions) with 9bp barcode sequences. Reverse transcription tagged the other adaptor on the 5′ end through template-switching activity. PCR amplification incorporated specific uracil-containing primers. The sticky end PCR fragments, generated by USER, were linked together with head-to-tail ligation. This produced ∼16 million fragments, which is 5–4 times more than traditional Pacbio sequencing (4 million). More details can be found in our previous research (19).

In this research, our goal was to generate 100 million cDNA reads at a low cost using nanopore high-throughput sequencing. To achieve this goal, we developed the FLOUR-seq platform, which combines the BD Rhapsody microwell system with nanopore sequencing (Figure 1A). Single cells were captured in microwells and labeled with beads containing 52 bp mega barcodes. The large Levenshtein distance among these barcodes compensated for the accuracy deficiency (70–90%) of nanopore sequencing (Figure 1D).

In previous studies, such as our own HIT-scISOseq and those by Cole et al. (2018), Goetz et al. (2012), Gierahn et al. (2017), and Zilionis et al. (2017), researchers tagged the same sequence on the 5′ end during reverse transcription (Smart-seq) (13) (46) (47) (48). However, this approach prevented the amplification of shorter fragments (less than 400 base pairs) with stem-loop structures (see Figure 1B). Although this technology offered the benefit of avoiding dimer amplification, it also presented challenges in observing the degrading short RNAs. To capture a complete picture of RNA expression, including nascent, mature, and short degrading RNAs, we used exonuclease I (ExoI) to remove excess primers on beads after reverse transcription. This method allowed us to avoid amplification of unwanted dimers. We then tagged the other 5′ adaptor on recycled beads (Figure 1C) and used PCR to efficiently amplify all types of cDNAs, which provided us with a comprehensive view of the transcriptomic landscape. As a result, the improved method could amplify more RNAs less than 400 base pairs. Finally, we could sequence the amplified cDNA on the nanopore platform.

We have also updated the HIT-scISOseq V2 protocol, which captures the RNA landscape of single cells on the 10x Chromium platform using a similar method. In comparison to FLOUR-seq, HIT-scISOseq on the 10x Chromium platform has cell barcodes with a lower Levenshtein distance (Figure 1D). The amplified cDNA obtained from HIT-scISOseq can be sequenced using PacBio, which can accurately differentiate between closely related barcodes.

In our updated version (HIT-scISOseq V2), we made two important changes to our protocol. Firstly, we modified the template switching primer. Traditionally, we and most others have used the same adapter sequence to create the stem loop structure, in order to prevent dimers and short sequence amplification. However, we have discovered that short sequences can still be valuable for velocity analysis. Therefore, we modified the 5′ end adaptor sequence to amplify a range of RNAs, including both long and short degraded RNAs. At the same time, we updated the purification process to include the short RNAs.

Secondly, we made changes to the ligation format. In the initial version, we proposed to randomly ligate cDNA amplicons, but this method led to various issues downstream. Consequently, we decided to modify the ligation format to 1–2–3–4. This new format requires four different PCRs, each tagging a distinct sequence at the end of the amplicon, in order to prepare the amplicon for downstream sequential ligation.

Comparing the FLOUR-seq and HIT-scISOseq with other methods, most of them rely on bioinformatic methods and NGS for their accuracy. One example of direct sequencing on single-cell RNAs is scCOLOR-seq, which uses duplex barcodes (ex. AATTGGCC) to correct barcodes in the bioinformatic method (see Figure 1I). However, creating the single-cell bead with duplex barcodes is difficult for most labs. HIT-scISOseq and FLOUR-seq are other methods that address this issue.

HIT-scISOseq uses the popular 10xGCC systems, but the downstream Pacbio sequencing may limit throughput to 16 million, which is still low for single-cell sequencing. FLOUR-seq, on the other hand, takes advantage of the BD Rhapsody three-barcode system with a large distance to efficiently demultiplex samples, which are then sequenced by the nanopore to generate around 67 million reads (Figure 1I). While the demultiplex efficiency is lower than that of scCOLOR-seq, FLOUR-seq is more accessible for normal labs. As our observation, the FLOUR-seq and HIT-scISOseq have the better sensitivity to detect gene features than the scCOLOR-seq in the same sequencing depth (lab Drop-seq based) (Figure 1J) (49). The price cost per million reads is much lower than the Sequel II but higher than the NGS based method (Figure 1K).

Both FLOUR-seq and HIT-scISOseq V2 are capable of capturing the RNA lifecycle (including nascent, mature, and degraded RNAs) in single cells. FLOUR-seq uses BD Rhapsody microwell system with nanopore sequencing, while HIT-scISOseq V2 uses the 10x Chromium platform with Pacbio sequencing.

### Validation and performance of FLOUR-seq

To accurately assign barcodes to cells, we used a computation strategy based on the bowtie-based pipeline, which is great for quickly calculating barcodes in large quantities (Figure 1E). We compared our strategy with a blastn-based algorithm (Figure 1F) and achieved similar results but with much faster speed. Using simulated data, our strategy recovered 65% of high-confidence barcodes with a barcode sequencing error rate of up to 10% (Figure 1G). This conservative approach only recovers high-confidence barcodes, resulting in a high precision of 0.968 (Figure 1F). A more aggressive method may be able to recover more barcodes with UMI correction (21). Afterward, UMIs were grouped and corrected at both the cell barcode and gene levels.

FLOUR-seq was validated using human HEK293T and mouse 4T1 single-cell BD libraries, each consisting of approximately 2400 cells in a 50:50 ratio. The libraries were then subjected to nanopore sequencing, with 65% of all reads showing complete alignment across 90% of the barcode sequence. Of these reads, 70% identified the presence of a poly(A) sequence.

After computational filtering, the nanopore sequencing identified 537 human cells, 658 mouse cells, and 5 collisions (Figure 1H). The Levenshtein distance between recovered cell barcodes was around 16, which theoretically tolerates > 30% sequencing error. The mixed species were most likely generated by cell loading.

We used HIT-scISOseqV2 (Pacbio-based, **3 flowcells**) and FLOUR-seq (ONT-based,**1 flowcell**) to conduct full-length single-cell RNA sequencing on a model of spermiogenesis. Both methods selected the top 3000 cells with a minimal count number 3616 (FlOUR-seq) and 3091 (HIT-scISOseqV2) (Supplemental Figure S1a, S2a). The average gene number in each cell were around 2263 and 2405, respectively, for both methods (Supplemental Figures S1b, S2b). For FLOUR-seq platform, the average transcript length was around 734 bp (Supplemental Figure S3a). The cells were grouped into 5 major subpopulations on UMAP plots (Supplemental Figures S1c, S2c), with similar numbers of each cellular population on both platforms, indicating the accuracy of cell barcode demultiplexing. The distribution of marker genes on cellular subpopulations (Dmrt1-spermatogonia, Piwil1-spermatocytes, Tex21-round spermatids, Tnp1-elongating spermatids, Cldn11-Sertoli cells, Fabp3-Leydig cells) was similar with both methods (Supplemental Figure S1cde, S2cde). The overall correlation of gene expression between the two methods was around 0.93, which is comparable to the conventional similarity between two sequencing platforms, suggesting accurate quantification(Supplemental Figure S3b). 63.3% of transcripts had at least 1 introns, and 43% of transcripts had <2 exons, likely reflecting RNA processing during biological development from nascent to degrading form (Supplemental Figure S3cd). All of this data was also observed on the Pacbio platform with a similar pattern (Supplemental Figure S3ef), reinforce the reliability of FLOUR-seq to identify the exon and intron. Since introns could be falsely generated due to PCR fusion artifacts, we used mixed-cell data to determine the frequency of false-positive fusion events. Mixed-cell data showed that only 1% of total reads contained fusion events, suggesting minimal influence of PCR fusion artifacts (25). Our results suggest that FLOUR-seq and HIT-scISOseqV2 can generate a reliable and high-throughput single-cell RNA life cycle.

### Progress of original RNA velocity with single cell RNA lifecycle

During development, cells differentiate over a period of hours to days, which is approximately the half-life of mRNA. By examining the levels of nascent (unspliced) and mature (spliced) mRNA, it is possible to estimate the rates of gene splicing and degradation. The concept of ‘RNA velocity’ proposes that similar signals may be discernible in single-cell RNA sequencing (RNA-seq) data. This could reveal changes in the entire transcriptome during dynamic processes, including their rate and direction (29) (30). RNA velocity quantifies the time-dependent relationship between the abundance of precursor (unspliced transcripts, u) and mature mRNA (spliced transcripts, s) by assuming a robust model for transcriptional dynamics, involving transcription rate (α), splicing rate (β=1), and the degradation rate (γ) of the spliced mRNA abundance, as determined by the steady state (Figure 2A). Next, they used the following equation to represent the unspliced transcripts and spliced transcript generation rate.


\begin{equation*}\frac{{du}}{{dt}} = \alpha - \beta *u\left( t \right)\end{equation*}

\begin{equation*}\frac{{ds}}{{dt}} = \beta *u\left( t \right) - \gamma *s\left( t \right)\end{equation*}

The production rate of unspliced transcripts du/dt is equivalent to the transcription speed α minus the spliced rate βu(t). Meanwhile, the rate of spliced form generation ds/dt is equal to the unspliced rate βu(t) minus the degradation speed γs(t).

RNA velocity introduces a sturdy model known as ‘steady state’ by assuming that the speed of nascent transcription (du/dt) and spliced transcription (ds/dt) is 0 when in a steady state (Figure 2B). The steady states are approached asymptotically when the rate of transcription α is constant and constrained to a fixed-slope relationship where β*u(t) = γs(t)* (default β=1) (Figure 2B). This occurs under the balance (the spindle shape) between the production of spliced mRNA from unspliced mRNA (β*u*) and mRNA degradation (*γs*). u > γs indicates induction of gene expression, while u < γs indicates repression of gene expression (Figure 2B). The gene-specific equilibrium coefficient γ is estimated through regression on the extreme expression quantiles, ensuring robust estimation even when most observed cells are outside of the steady state (29). The extrapolated state can also be estimated with α and γ (29).

Due to the limitations of short-read sequencing, most researchers only differentiate between the ‘u’ and ‘s’ forms from the partial sequence close to the 3′ UTRs (Figure 2C). However, our developed Pacbio-based HIT-scISOseq V2 and nanopore-based FLOUR-seq revealed a significant underestimation of unspliced transcripts for most genes in short-read sequencing (24.1% versus 8%) (Figure 2C). This discrepancy in unspliced counts can create challenges when performing RNA velocity analysis.

**Figure 3.**
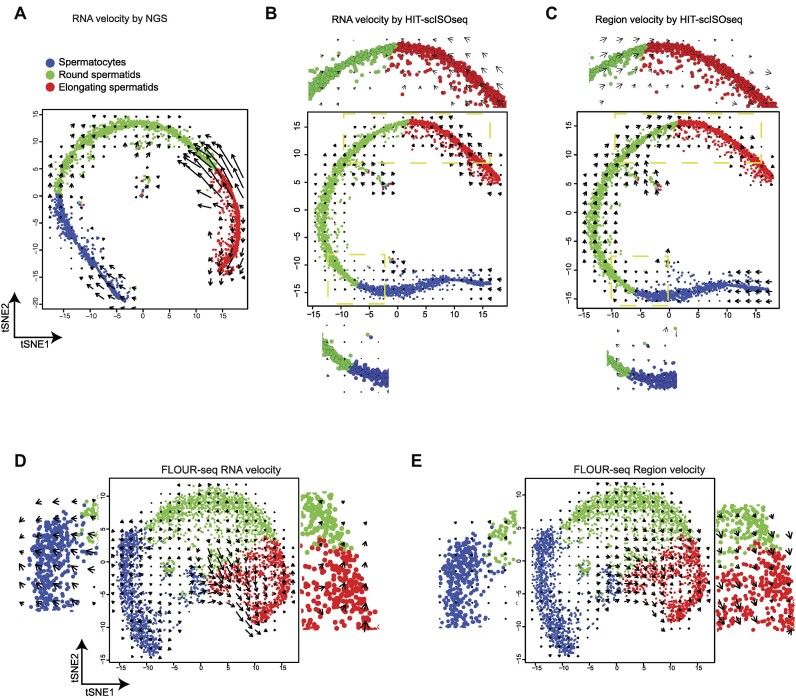
The velocity projection of spermiogenesis development. (**A**) Velocity field projected onto a tSNE plot of RNA velocity based on the NGS scRNA-seq. (**B**) The velocity field is projected onto a tSNE plot of RNA velocity (u/s) using the HIT-scISOseqV2 from full-scale scRNA data of mouse testicular cells (*n* = 3001 cells). Arrows indicate the average speed on a defined grid (number of grids = 20). Blue points represent spermatocytes, green points represent round spermatids, and red points represent elongating spermatids. We magnify the cell differentiation key stages for observation - from spermatocytes to round spermatids and from round spermatids to elongating spermatids. (**C**) Velocity field projected onto a tSNE plot of region velocity (intron/exon) based on the HIT-scISOseqV2 from full-scale scRNA data of mouse testicular cells (*n* = 3001 cells). (**D**)Velocity field projected onto a tSNE plot of RNA velocity (u/s) based on the FLOUR-seq from full-scale scRNA data of mouse testicular cells (*n* = 3001 cells). Arrows indicate the average speed on a defined grid (number of grids = 20). Blue points represent spermatocytes, green points represent round spermatids, and red points represent elongating spermatids. (**E**) Velocity field projected onto a tSNE plot of RNA velocity (intron/exon) based on the FLOUR-seq from full-scale scRNA data of mouse testicular cells (*n* = 3001 cells).

We tested this hypothesis by using a well-established biological model of spermatogenic development (Figure 3A, B). To obtain the velocity vector fields projected onto the t-distributed stochastic neighbor embedding (tSNE) plot, we applied the original framework of RNA velocity and used the s and u forms from the 10x scRNA-seq (NGS) data and HIT-scISOseq V2 data, respectively. This approach allowed us to observe differences in the tSNE dimension of cell clustering and cell fate indicated by vector fields. However, despite these efforts, both methods failed to produce the expected sequence of biological development during spermiogenesis.

### The region velocity concept

Upon analysis, we discovered that the unspliced transcripts lacked specificity, and that the unspliced transcripts with different introns yielded the same unspliced counts. Given the detailed data generated by HIT-scISOseqV2/FLOUR-seq, we intend to expand the RNA velocity model beyond its original framework, utilizing more robust parameters generated by long reads sequencing technology such as transcript length and intron/exon count.

SLAM-seq (thiol-linked alkylation for the metabolic sequencing of RNA) is a metabolic labeling technique that enables the observation of changes in nascent RNAs over time (50). It specifically measures changes in RNA synthesis rates over a period of 10–100 min for most genes. Among the measured parameters, we observed that the intron/exon ratio and unspliced/spliced ratio were significantly higher (4–5× fold) in nascent labeled RNAs compared to matured RNAs, while we found no substantial difference in transcript length (Figure 2D). Additionally, we noticed that the difference (fold change) in the exon/intron ratio gradually decreases over 4sU labeling time as more RNAs are labeled and spliced into mature RNAs (Figure 2D). This suggests that the ratio correlates with the progress of RNA processing.

It is worth noting that the intron/exon ratio displays less variation than the unspliced/spliced ratio, indicating that it may be a more dependable parameter (Figure 2D). Additionally, the exon/intron ratio was found to be more stable and better suited for steady-state modeling when compared to unspliced/spliced counts. This intron/exon dynamics results in a more streamlined spindle shape, making it more accurate in estimating γ (the slope) and more appropriate for extrapolating future rates of RNA synthesis (Figure 2E).

To put it simply, a higher correlation between spliced/exon counts and unspliced/intron counts in different cells indicates that the cells are closer to the steady-state line (fitted line) and have a more reasonable projection onto the state of the next time point obtained using steady-state model parameters. However, the correlation coefficients between the ratio of unspliced to spliced cells were notably lower than those of intron/exon counts. In fact, over 55.9% of genes had an intron/exon correlation coefficient greater than 0.4 (Figure 2F). This allowed for more genes to be included in downstream velocity analysis. This was necessary due to the abundance of zero values of spliced counts that prevented the calculation of their correlation with unspliced counts (Figure 2F) (30). Using this process, intro/exon counts were substituted for spliced/unspliced counts in RNA velocity, resulting in a more robust steady-state model.

The main hypothesis of the model is similar to that of RNA velocity. The change in exons and introns during the RNA transcription dynamics process is proportional to the change in unspliced and spliced counts in RNA velocity. Typically, exon changes only occur during transcription (increase) and degradation (decrease), while intron changes only occur during transcription (increase) and splicing (decrease). Therefore, by the law of mass action, the rate equations for each gene, regardless of which, are expected to have evolving exons count e and introns count i over time, which can be simply described as follows:


\begin{equation*}\frac{{di}}{{dt}} = \ {\alpha }_i\left( t \right) - \beta *i\left( t \right)\end{equation*}

\begin{equation*}\frac{{de}}{{dt}} = \ {\alpha }_e\left( t \right) - \ \gamma *e\left( t \right)\end{equation*}

where, *α_i(t)_*and *α_e(t)_*are the transcription rate of *i* and *e* respectively. β is the splicing rate and γ is degradtion rate. However, *α_i(t)_*and *α_e(t)_* have specific relations as the nascent mRNA produced during transcription process are consist of introns and exons. So, we introduce a parameter - portion of introns in nascent mRNA (*θ*) as this parameter can be estimated more easily from observed data than *α_i(t)_*and *α_e(t)_*directly. Then equations can be simplified as follow:


\begin{equation*}\frac{{di}}{{dt}} = \theta *\alpha \left( t \right) - \beta *i\left( t \right)\end{equation*}

\begin{equation*}\frac{{de}}{{dt}} = \left( {1 - \theta } \right)*\alpha \left( t \right) - \gamma *e\left( t \right)\end{equation*}

Here, *θ* represents the proportion of introns *N_intron_/(N_intron_+ N_exon_)* involved in the transcription process, while *1-θ* represents the proportion of exons. *α(t)* indicates the transcription rate, and *e(t)* and *i(t)* represent the numbers of exons and introns at time point *t*, respectively. *β* (default = 1) and γ are the splicing and degradation rates. Therefore, *di/dt* and *de/dt* describe the speed of intron and exon changes, respectively. We call this process of state change ‘Region Velocity’ because the time derivatives of the numbers of intragenic and transcribed regions can be used to infer the state of the cell throughout the process.

We used the basic steady-state model (refer to supplementary notes) to calculate the unknown parameters (α, β, γ, and θ). At this point, the exon and intron reach a balanced state where de/dt and di/dt are both zero, which is similar to RNA velocity. Additionally, we conducted a formula transformation.


\begin{equation*}\theta *\alpha \left( t \right) = i\left( t \right)\end{equation*}

\begin{equation*}\left( {1 - \theta } \right)*\alpha \left( t \right) = \gamma *e\left( t \right)\end{equation*}

\begin{equation*}\gamma = \left( {\frac{{i\left( t \right)}}{{e\left( t \right)}}} \right)*\left( {\frac{{1 - \theta }}{\theta }} \right)\end{equation*}

Previous research has shown that the slope *i(t)/e(t)* can be calculated by robust extreme quantile regression (29), allowing for the solvability of gamma and alpha. Data simulation has revealed that intron/exon levels above or below the identity line could indicate the induction and repression statuses, respectively (Figure 2B).

### Region velocity predicted the spermiogenesis development

In the HIT-scISOseq-V2 dataset of sperm cells, we observed that the vector fields projected onto the tSNE embedding plot of the steady-state model (Figure3c) indicated that the direction of flow of the region velocity was more aligned with the expected spermatogenic waves. Specifically, there was a streamlined flow from spermatocytes to elongating spermatids.

**Figure 4.**
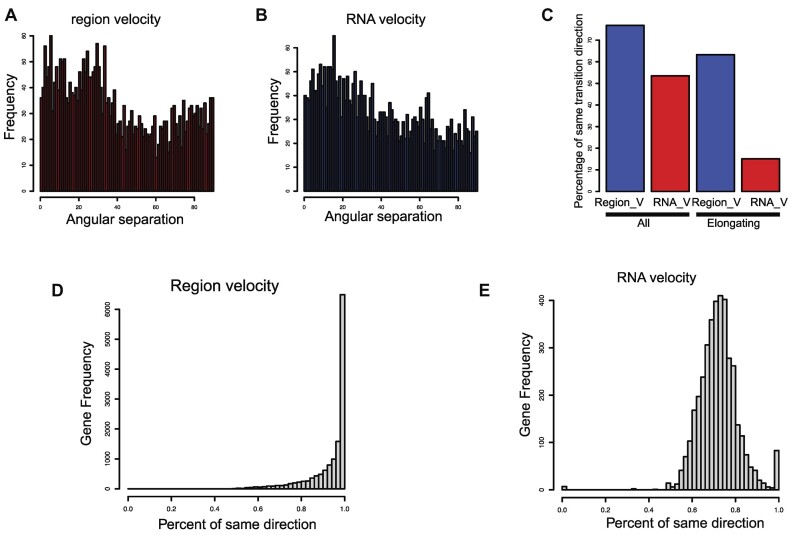
Quantitively analyze the velocity performance. (**A**) Measure the angular separation in region velocity. The pattern of spermiogenesis development is a directional lined pattern that progresses in one direction. In order to estimate the observed directions, we use regression on groups of neighboring cells. The angular separation is then measured as the angle between the observed and predicted directions. (**B**) Measure the angular separation in RNA velocity. (**C**) If the angular separation is less than 90 degrees, the predicted and observed directions tend to be similar. If the angular separation is greater than 90 degrees, it means they are pointing in different directions. To compare the accuracy of the predicted directions based on the angular separation, this bar chart summarized the results to show the percentage of predicted arrows pointing in the same direction as the observed direction. ‘All’ refers to all cell predictions, while ‘Elongating’ refers to cell predictions in the elongating stage. (**D**) The distribution of direction difference between observation and prediction results spanning all genes in Region velocity. The increase/decrease tendency of exons between observed cells and predicted cells was compared with the tendency of exons between observed cells and its pseudotime-depended nearby observed cells. The histogram showed the distribution of matched hits percentage for each gene across all cells. (E) The similar method was performed with the RNA velocity.

When compared to velocity calculations based on RNA abundance, which measure both unspliced and spliced molecules (Figure 3A, B), region velocity calculations more accurately reflect the transformation of spermatocytes into round spermatids and elongating spermatids (as shown in the magnified scope in Figure 3A–C). We then measured the difference between the predicted and expected direction of movement, with a smaller angular separation indicating better alignment with the expected direction. The regional velocity method showed significantly better performance than RNA velocity (Figure 4A, B), particularly in elongating spermatids. In fact, region velocity successfully predicted the expected direction in 62% of cells, while RNA velocity only predicted it in 13% (Figure 4C). Additionally, when we compared the predicted gene expression in the next stage with the observed gene expression in the close stage, most of the genes predicted by region velocity showed a tendency to increase or decrease that matched the observation, with a success rate of over 80% (Figure 4D). RNA velocity only demonstrated a success rate of 0.6–0.8 in matching observed gene expression (Figure 4E). Overall, the region velocity method demonstrates superior performance.

Additionally, the elongation period of the spermatids cluster lasted longer, as evidenced by the increased speed at the round stage and decreased speed between the round phase and the start of elongation. Therefore, region velocity calculations suggest that meiosis and elongation are the main bottlenecks in spermatogenesis, and their effects are similar based on the size of velocity field lengths. This is evident from the deceleration of RNA velocity at the end of the process of transformation of spermatocytes into round spermatids, which is consistent with increased RNA transcription at the beginning of the process and during long periods of the two-step meiosis stage (51).

Based on previous research (30), our team has identified driver genes that play a crucial role in the development of spermiogenesis (refer to Supplemental Table 1). These genes were also found to be enriched in reproductive functions through Gene Ontology (GO) term analysis (Supplemental Figure S4d). The contribution of these genes to velocity prediction was identified in a spindle shape, indicating that they had a greater impact on the prediction (Supplemental Figure S4ab). We quantified this spindle distribution as likelihood (Supplemental Figure S4c). In addition, we observed that the intron exon change of driver genes was accompanied by splicing dynamics (Supplemental Figure S5a). We summarized the splicing types of these driver genes and found that most splicing decreased during spermiogenesis, with only a few increasing the splicing frequency. As examples, we chose Taf9, Suv39h2, and Tor1aip1 to demonstrate the different isoform changes, and we found that these isoforms had tissue specificity (Supplemental Figure S5b).

In addition to driver genes, we have identified genes that are involved in degradation (Supplemental Figure S6). We observed that genes related to mitochondria, ATP generation, and ribosome assembly were downregulated during the spermiogenesis process. These findings are in line with previous research on mitochondrial fusion and fission during spermiogenesis (52).

It is worth noting that the third-generation FLOUR-seq platform utilizes nanopore sequencing technology, while HIT-scISOseq V2 relies on PacBio sequencing. Our research suggests that region velocity is more applicable to FLOUR-seq data compared to conventional RNA velocity. In fact, the region velocity was able to successfully predict the developmental flow, unlike the conventional RNA velocity (Figure 3D, E). Additionally, the intron/exon parameters remain robust and are not significantly affected by potential errors introduced by error-prone sequencing methods. This is important as such errors may lead to misclassification of splice and unspliced transcription. However, the large number of intron/exon parameters can tolerate the impact of these errors (Supplemental Figure S4e). As anticipated, the region velocity for FLOUR-seq also generated the expected sequence of spermiogenesis development.

In conclusion, our research has revealed the significance of certain driver genes in spermiogenesis, and has demonstrated the usefulness of region velocity in analyzing HIT-scISOseq V2/FLOUR-seq data. Our results offer valuable perspectives for forthcoming studies on predicting cell fate.

### Region velocity applied to the short reads sequencing

While nanopore sequencing has advantages, it may not always meet research requirements for detecting rare genes and cell types through deep sequencing. To address this issue, a recent technology called VASA-seq uses short-read sequencing to assemble full-length RNAs in single cells (45). VASA-seq fragments the RNA in a cell, tails the RNA with a polyA tail, and then performs reverse transcription and T7 amplification. While VASA-seq is not a perfect solution, it does fill the gap for ultra-high throughput requirements and can detect whole transcription, including with or without a polyA tail.

Despite detecting more genes than HITscISOseqV2 and FLOUR-seq (Supplemental Figure S7a), VASA-seq identified fewer introns and unspliced transcripts than FLOUR-seq (Supplemental Figure S7b). However, VASA-seq detected twice as many introns as conventional scRNA-seq, which improved the intron detection sensitivity of NGS. Region velocity offers better durability for intron detection bias, as it only counts the number of introns and exons without assembly. Therefore, region velocity is well-suited to VASA-seq because single-cell sequencing makes it difficult to fully assemble the entire transcriptome, and some assembly may be mistaken. By comparing embryonic stem cells at E8.5 and E9.5, region velocity showed the correct trend in directing cell development from E8.5 to E9.5 (Supplemental Figure S7c).

In conclusion, the FLOUR-seq platform has been demonstrated to be a reliable and high-throughput method for capturing the RNA lifecycle in single cells. The validation of FLOUR-seq using human HEK293T and mouse 4T1 single-cell BD libraries, as well as its use in a model of spermiogenesis, has shown its potential for the study of dynamic biological processes. Moreover, the region velocity approach utilizing intron/exon counts has demonstrated superior performance in predicting cell fate across different species and cell types compared to conventional RNA velocity.

## Discussion

We present the development of the FLOUR-seq platform, which combines the BD Rhapsody microwell system with nanopore sequencing to generate 100 million cDNA reads at a low cost. The platform was validated using human HEK293T and mouse 4T1 single-cell BD libraries, and was found to be capable of capturing the RNA lifecycle in single cells. Additionally, we discuss the use of the HIT-scISOseq V2 protocol to capture the RNA landscape in single cells on the 10x Chromium platform using a similar method. Finally, we conclude with a discussion of the region velocity approach, which uses intron/exon counts to more accurately predict cell fate.

HIT-scISOseq V2 and FLOUR-seq are two types of single-cell RNA sequencing technologies that can capture the entire RNA profile (including nascent, mature, and degraded RNAs) in individual cells. HIT-scISOseq V2 utilizes the 10× Chromium platform with PacBio sequencing, while FLOUR-seq employs the BD Rhapsody microwell system with nanopore sequencing. FLOUR-seq can also expand to any three barcode system with a large distance such as the DNBelab C4 single-cell lab. HIT-scISOseq V2 boasts a higher barcode sequencing accuracy but a lower cDNA read count, while FLOUR-seq has a lower barcode sequencing accuracy but a higher cDNA read count. Nanopore sequencing has a higher error rate, which can sometimes lead to misidentification of the intron/exon or unspliced form. Nevertheless, this high error rate did not affect the region velocity analysis. A few other algorithms were necessary to accurately identify the isoforms, but this could introduce some bias. Both of these technologies have been validated and used to study spermiogenesis.

Nanopore sequencing presents a challenge due to potential errors in barcode sequencing, which can hinder the accurate demultiplexing of cells. To overcome this challenge, the FLOUR-seq platform incorporates large (52 bp) barcodes with a high Levenshtein distance, which compensates for the error rate of nanopore sequencing. During this process, low-quality reads and cell barcodes with a difference of less than 4bp are discarded. Additionally, a computation strategy that uses a bowtie-based pipeline is employed to quickly assign barcodes to cells. Based on simulation data, this strategy is capable of recovering up to 65% of high-confidence barcodes with a barcode sequencing error rate of up to 10%. However, 35% of the reads are still wasted. Other bioinformatics laboratories may be able to further rescue these reads and improve the UMI quantification by employing more aggressive algorithms.

The data from HIT-scISOseq/FLOUR-seq is highly applicable in the region velocity model. Recently, Bergen *et al.* proposed a dynamic model to more accurately infer the parameters because many genes have a discrete distribution other than the spindle model (30). Although the dynamic model of RNA velocity is more rigorous in parameter inference, our focus was on the steady-state model, which is more straightforward for establishing and studying new models despite its less rigorous nature. We also developed dynamic model in region velocity using EM algorithm (see supplemental note). However, because many of the intron/exon are distributed as the spindle shape with high correlation coefficient, the dynamic model did not significantly improve the region velocity.

RNA velocity is a technique used in single-cell sequencing to analyze how gene expression changes over time. However, this approach has limitations when using short-read sequencing. It can incorrectly identify intron and splicing isoforms, which can be problematic. To address this issue, Gao, M et al. developed UniTVelo (53), a technique that uses a profile function of spliced RNAs and the Gradient Descent algorithm to infer the parameters. Traditional RNA velocity is based on a linear model through steady state of gene expression over time. To capture the nonlinearity of RNA velocity dynamics, Zhanlin Chen *et al.* developed deepVelo (54). They trained a variation autoencoder to predict continuous changes of gene expression in an individual cell over time. Li *et al.* proposed a relay velocity model in single-cell resolution to predict RNA velocity of each gene in each cell through training a deep neural network model for each gene (55). As a result, the resolution and accuracy of RNA velocity continue to improve, which is the direction of its development.

However, the development of RNA velocity is still based on the traditional model with short-read sequencing, which relies on spliced and unspliced forms. The main limitation of this development direction is the NGS sequencing platform, which cannot produce abundant observations and mainly focuses on gene expression like spliced counts or unspliced counts. In our study, we used Pacbio and ONT sequencing platforms, which provide more observations, and developed region velocity based on observations of exons and introns using different ordinary differential equations. This approach differs from the traditional model.

Nevertheless, the development of RNA velocity has also inspired us on how to improve the performance of region velocity. In the future, using deep neural network methods or other machine learning methods to improve accuracy and robustness will be an available choice.

Compared to previous studies on RNA velocity, HIT-scISOseq/FLOUR-seq can gather more mRNA-related data such as full length and structure as observations, resulting in more indicators of cell state than just RNA abundance alone. We maintained the assumption of constant transcription rates, splicing rates, and degradation rates from previous work while inferring the regional velocity model. This inspired similar assumptions that the intron ratio of the transcription rate is constant and could be solved by a linear model. However, these assumptions may be undemanding and future iterations of the regional velocity model could increase the complexity of these assumptions or estimate them more accurately through experimental methods (56). However, the computation speed of regional velocity is slower because of one more parameter to be estimated. Nevertheless, because the steady-state models of regional velocity are estimated by linear fitting, the influence of low speed could be reduced by increasing the parallel computation resources.

The successful establishment of the regional velocity model exhibits referential significance for cell fate studies using other observations of HIT-scISOseq/FLOUR-seq. Regional velocity is essentially an upgrade of a new concept in an old framework because the core kinetic model from transcription to degradation remains unchanged. In fact, regional velocity could smoothly simulate the spermatogenic process from spermatocytes to elongating spermatids because the velocity model happens to be a time-dependent process while the process of spermatogenic waves is also related to time series.

However, the time variation process of mRNA might be more complex as our HIT-scISOseq/FLOUR-seq data observed that some ‘mRNA’ only contained introns and the intron length of the same mRNA remained the same, but the exon length reduced in contrast, indicating that the process from transcription to degradation of mRNA might violate the streamlined time order. More observation data provided by HIT-scISOseq/FLOUR-seq might discover new core kinetic models of RNA dynamics in future studies, which could provide better cell fate prediction for different species and cell types.

## Supplementary Material

gkad969_Supplemental_Files

## Data Availability

The data underlying this article are available in CNGB Sequence Archive (CNSA) of China National GeneBank DataBase (CNGBdb) under accession number CNP0004694, and NCBI Sequence Read Archive (SRA) BioProject under accession number PRJNA1010954. The experimental protocol (FLOUR-seq), and the HIT- scISOseq protocol, are available via: https://doi.org/10.17504/protocols.io.yxmvmnpm6g3p/v1. The protocol of region velocity pipeline is available at https://doi.org/10.17504/protocols.io.eq2lynejrvx9/v1. The bioinformatics pipeline of FLOUR-seq is available at https://doi.org/10.6084/m9.figshare.24258568.v1. The R package of Region velocity is available at https://doi.org/10.6084/m9.figshare.24258526.v1.
