# Supplementary material for: Improving the RNA velocity approach with single-cell RNA lifecycle (nascent, mature and degrading RNAs) sequencing technologies
Source: Nucleic Acids Res. 2023 Nov 6;51(22):e112. doi: 10.1093/nar/gkad969 (PMC10711548; doi:10.1093/nar/gkad969)
Supplement: gkad969_Supplemental_Files [file gkad969_supplemental_files.zip › Supplemental Figures 773aa4eca1ef491aa7090f198f711d79.pdf]

# Improving the RNA velocity approach with single-cell RNA lifecycle (nascent, mature and degrading RNAs) sequencing technologies

Chen Zhang[1, *], Yitong Fang[1, *], Weitian Chen[1,2,*], Zhichao Chen[1,*], Ying Zhang[3], Yeming Xie[1], Wenfang Chen[1], Zhe Xie[1], Mei Guo[1], Juan Wang[1], Chen Tan[1] ,Hongqi Wang[1], Chong Tang[1]

[1.] BGI Shenzhen, China, 518000
[2.] BGI Education Center， University of Chinese Academy of Sciences， Shenzhen 518083， China
[3.] Guangdong Provincial Reproductive Science Institute (Guangdong Provincial Fertility Hospital), Guangzhou, China;NHC Key Laboratory of Male Reproduction and Genetics, Guangzhou, China

[*] These authors contributed equally to this work.

Keywords: single-cell, ONT full-length sequencing, three-barcode technology, single cell maps, RNA velocity

Correspondence:

Chong Tang

Director of technology, BGI Shenzhen, China

Phone: 8618025420976

Email: tangchong@bgi.com

# Supplemental Figures

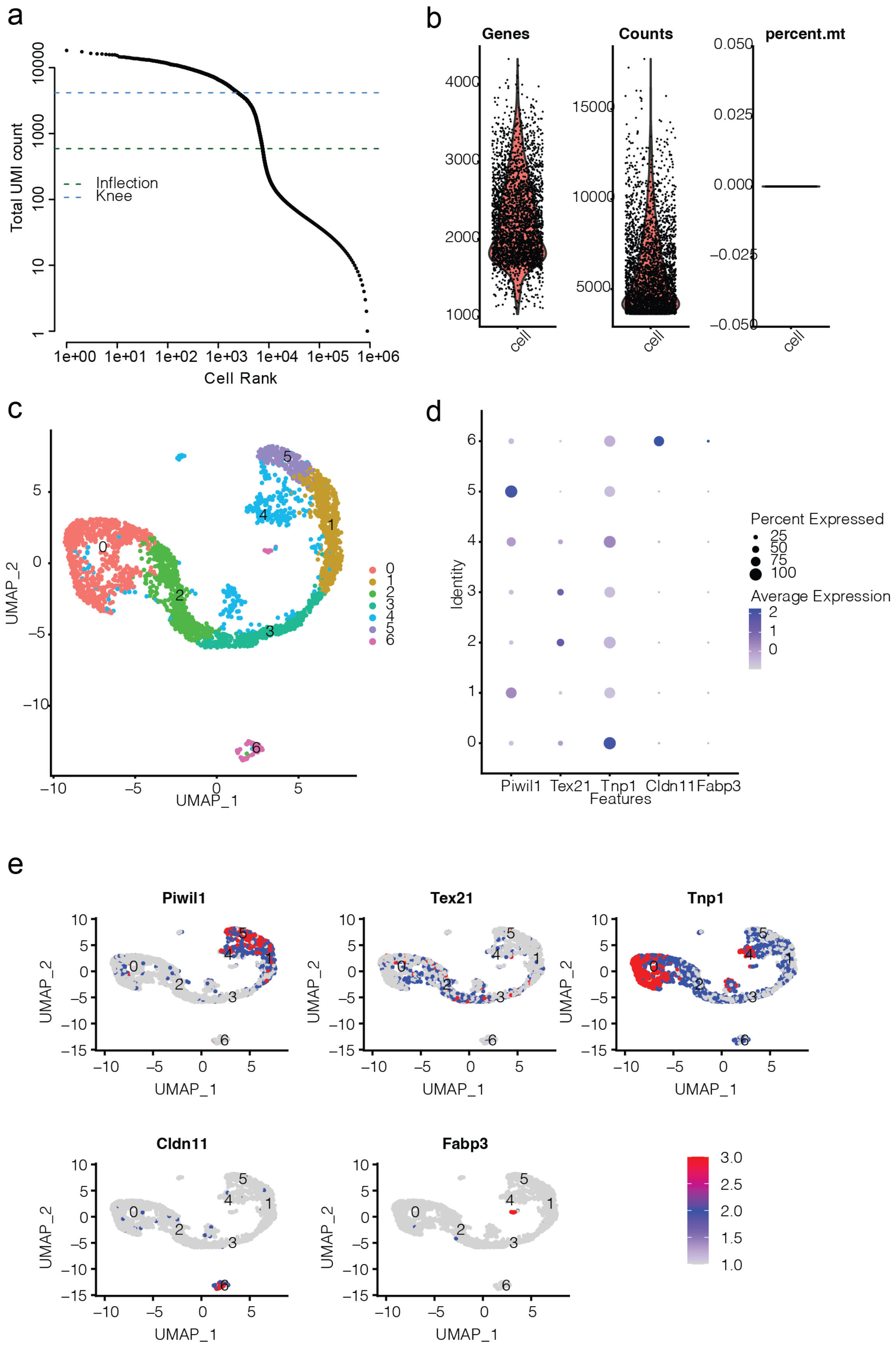

a
Total UMI count
Inflection
Knee
Cell Rank
b
Genes
Counts
percent.mt
cell
c
UMAP_2
UMAP_1
d
Identity
Percent Expressed
Average Expression
Piwil1 Tex21 Tnp1 Cldn11 Fabp3
Features
e
Piwil1
Tex21
Tnp1
Cldn11
Fabp3

**Supplemental Figure 1 displays the results of FLOUR-seq analysis on mouse spermary cells.**

(a) The elbow plot was used to determine the number of cells. (b) The density of gene numbers and gene counts in each cell is shown with Percent.mt representing the percentage of mitochondrial genes. (c) Cell clusters are plotted onto the UMAP plot with a total of 3,001 cells. (d) The dot plot shows marker genes including *Piwil1* (spermatocytes), *Tex21* (round spermatids), *Tnp1* (elongating spermatids), *Cldn11* (Sertoli cells), and *Fabp3* (Leydig cells). (e) The feature plots are displayed onto the UMAP of marker genes.

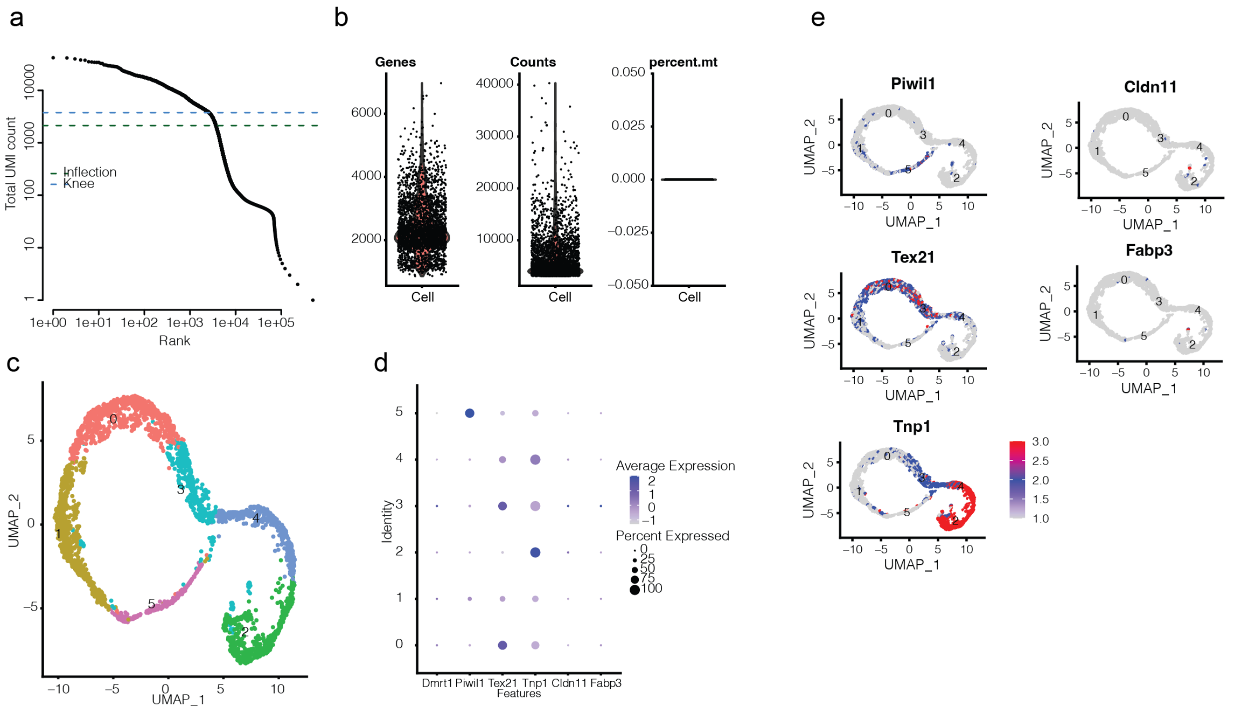


**Supplemental Figure 2 shows the performance of HIT-scISOseqV2 on mouse spermatocyte cells.** The plot in (a) displays the number of cells for each elbow point. In (b), the density of gene numbers and gene counts in each cell is shown, with the percentage of mitochondrial genes indicated as Percent.mt. Cell clusters are represented in the UMAP plot in (c), based on 3,001 cells. The dot plot in (d) visualizes marker genes, including *Dmrt1* for spermatogonia, *Piwil1* for spermatocytes, *Tex21* for round spermatids, *Tnp1* for elongating spermatids, *Cldn11* for Sertoli cells, and *Fabp3* for Leydig cells. Lastly, (e) features the plots of marker genes on the UMAP.

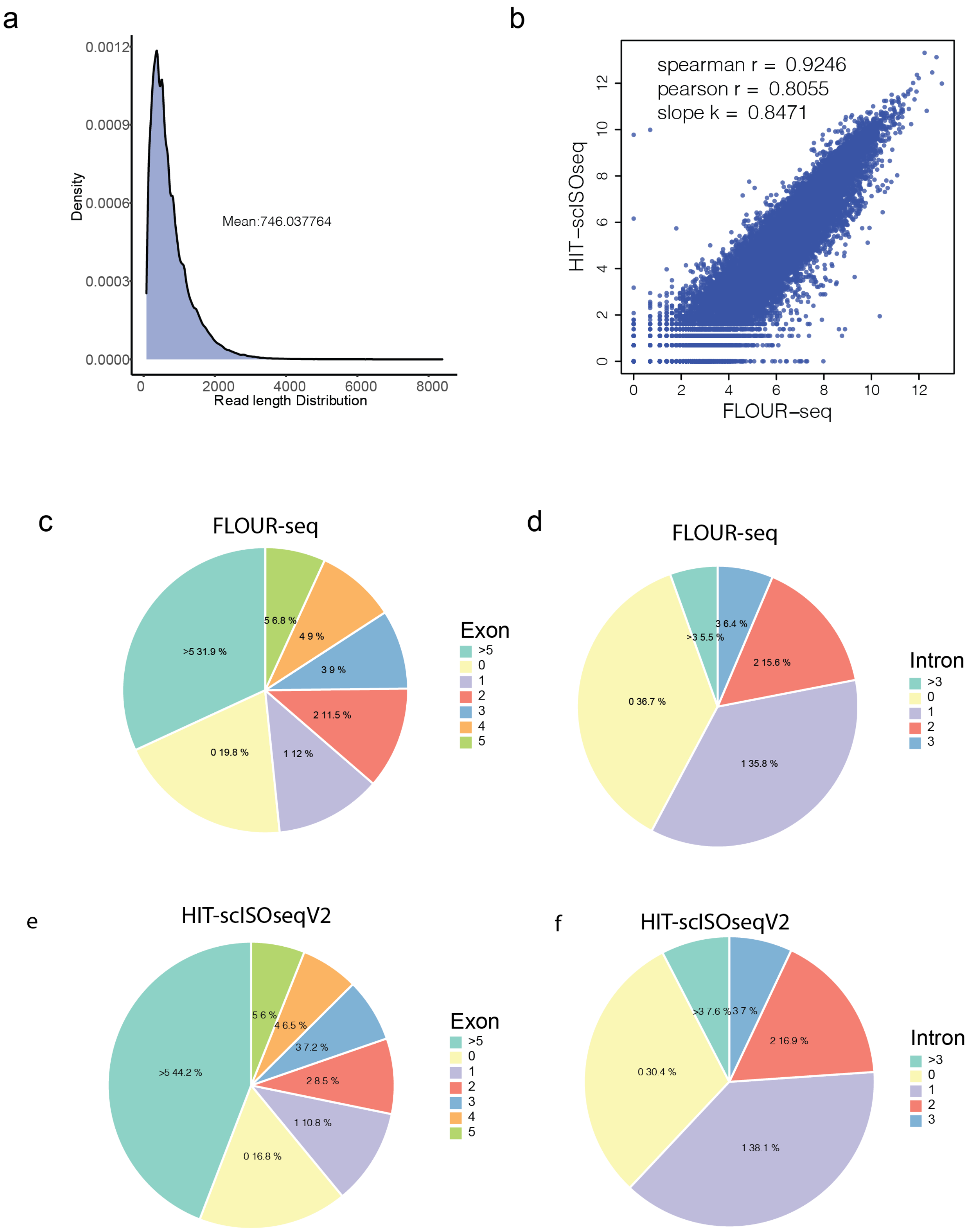


**Supplmental Figure 3. The parameters used in the velocity analysis.**

(a) The length distribution of the FLOUR-seq from the sequencing data.

(b) The gene quantificiaton correlation between the HIT-scISOseq and FLOUR-seq with pooling of all the single cell as bulk sample.

(c-d) The exon and intron distribution of FLOUR-seq. The color indicated the intron/exon number per transcripts.

(e-f) The exon and intron distribution of HIT-scISOseqV2.The color indicated the intron/exon number per transcripts.

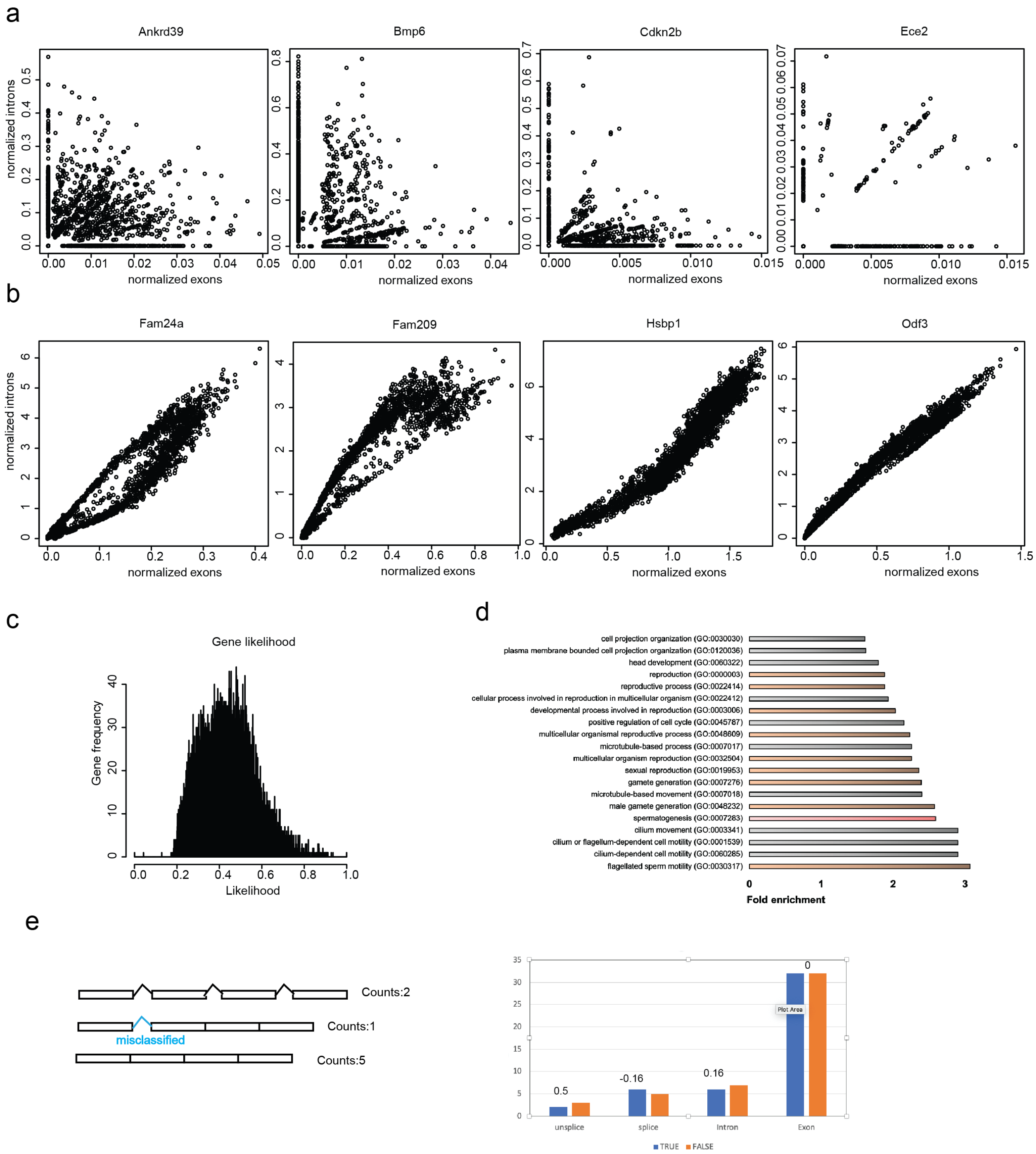


## Supplemental Figure 4. Driver Genes in Spermiogenesis.

(a) This figure displays the intron and exon counts of genes (Ankrd39, Bmp6, Cdkn2b, Ece2) in each single cell. Each dot represents a cell, while the X and Y axes represent the normalized exon and intron counts of one gene. These genes have a low likelihood of spindle distribution.

(b) The intron and exon counts of genes (Fam24a, Fam209, Hsbp1, Odf3) in each single cell are shown. Each dot represents a cell, while the X and Y axes represent the normalized exon and intron counts. These genes also have a high likelihood of spindle distribution.

(c) The likelihood distribution of spindle-shaped genes suggests how likely the distribution is to have a spindle shape.

(d) This panel displays the enrichment analysis of Gene Ontology terms for the top 1000 genes with the highest likelihood.

(e) The left panel displays a simulation of the errors that occur during the intron classification process. The right panel shows the spliced/unspliced intron/exon counts based on correct and incorrect identification. The number above the bar indicates the change if one intron is identified as a mistake.

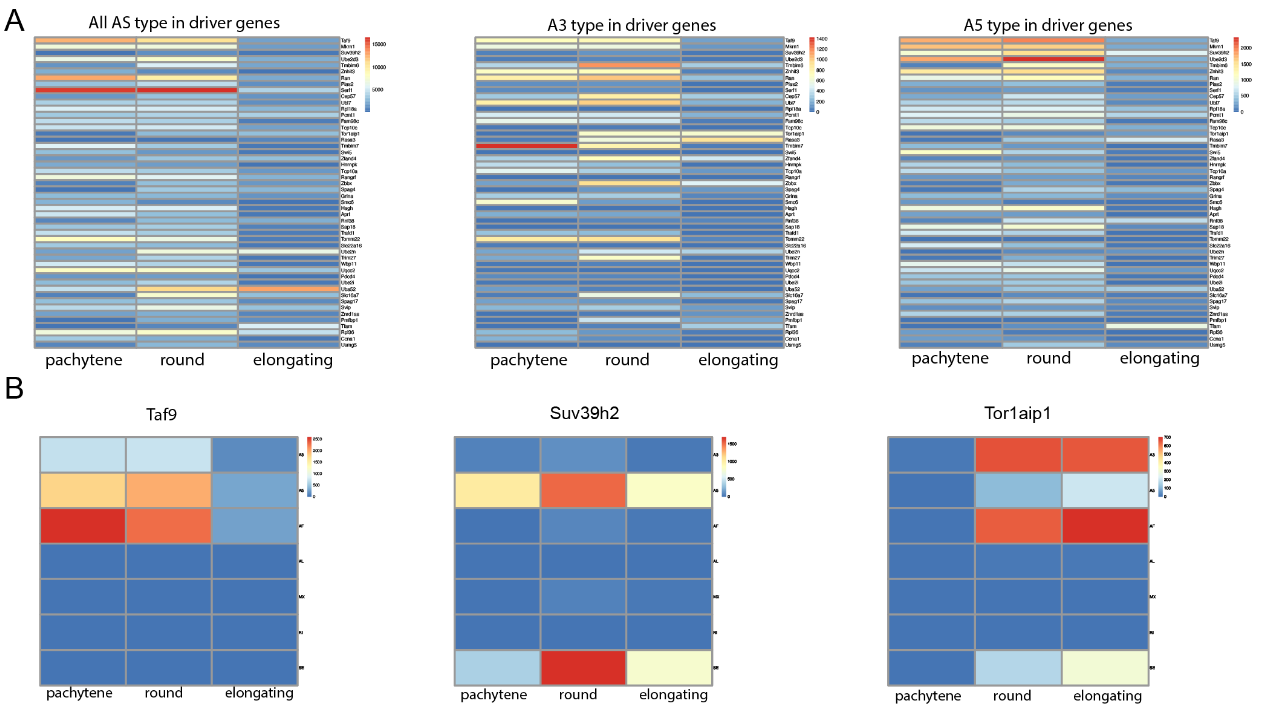


**Supplemental Figure 5. Dynamic splicing of the driver genes.**

(A) The splicing event counts were recorded for pachytene spermatocytes, round spermatids, and elongating spermatids. All AS types, including common splicing types such as intron insertion and exon skipping, etc. were included. The A3 type indicated

splicing events on the 3' UTR, while A5 indicated splicing events on the 5' UTR. The color indicated the number of event counts.

(B) The heatmap displays the isoforms present in each gene, Taf9, Suv39h2, and Tor1aip1. Each line in the heatmap represents a specific isoform in the gene, while the color indicates its abundance in pachytene spermatocytes, round and elongating spermatids.

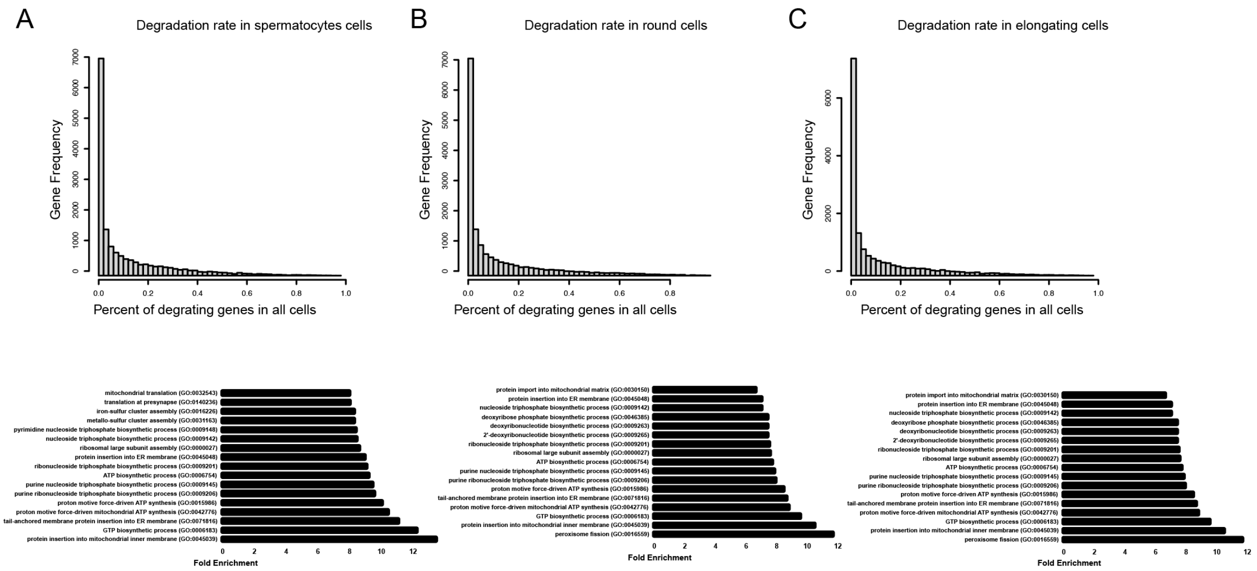


**Supplemental Figure 6. The degrading gene in spermiogenesis.**

(A) Based on our algorithm, we used the steady state to categorized the gene state in the cells, the induction state or degradation state. Then for this gene, we caculate how many cells in degradation state (x-axis, percent of cells with degradating status) and summarized the frequency of the genes in different cellular state composition. Then we selected >0.6 meaning more than 60% cells have this gene in degradation state to do GO term analysis.

(B) Degrading status in round spermatids.

(C) Degrading status in elongating spermatids.

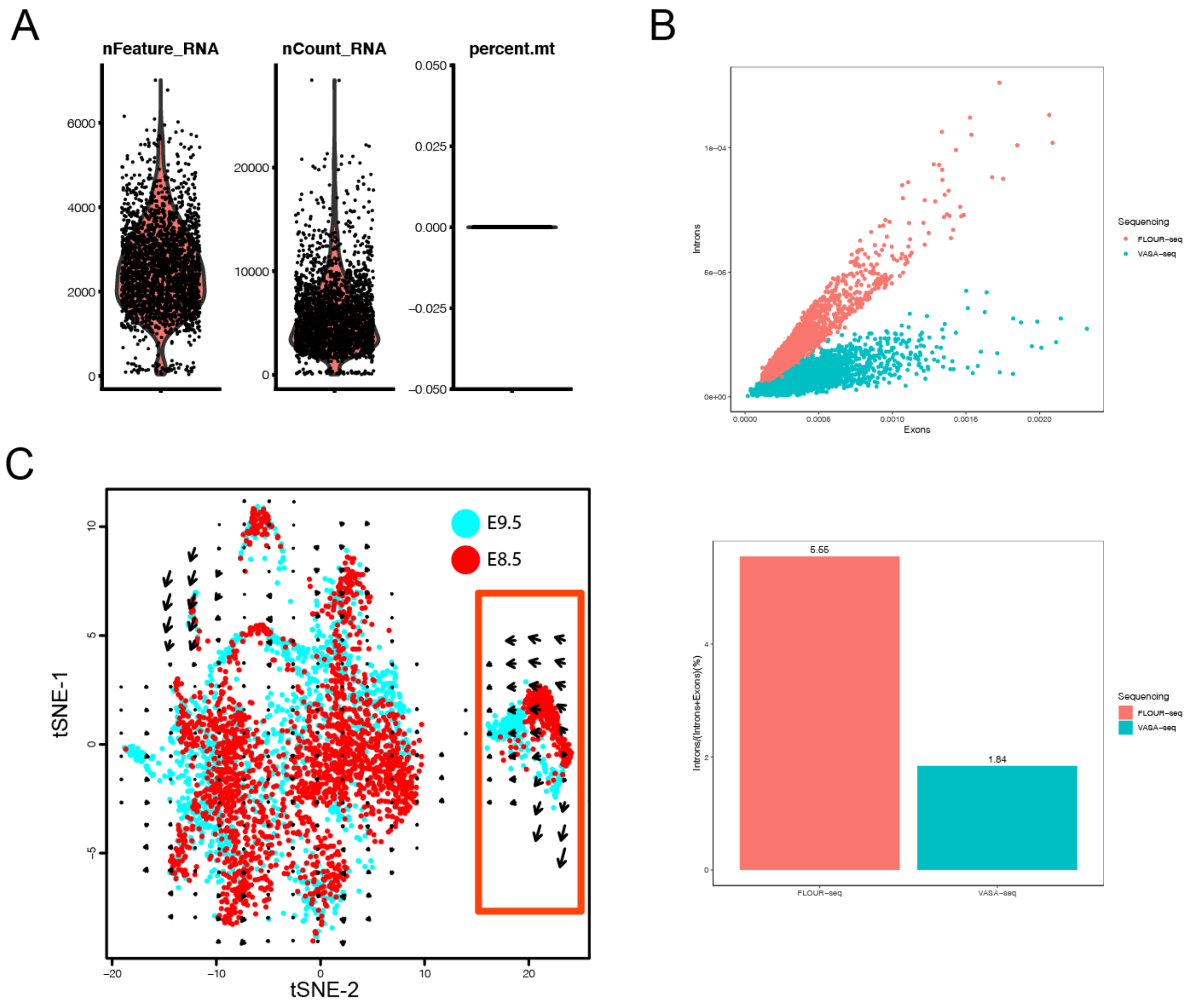


**Supplemental Figure 7. The region velocity on VASA-seq.**

(A) The density of gene numbers (nFeature_RNA) and gene counts (nCount_RNA) in each cell is shown, with Percent.mt representing the percentage of mitochondrial genes.

(B) The scatter plot showed the intron and exon in every cells. The intron and exon number normalized by the all intron plus exon number from all the cells. The bar plot showed the summarized intron/(intron+exon) from all cells. The red color indicated the FLOUR-seq and the blue color indicated the VASA-seq.

(C) The velocity field is projected onto a tSNE plot of region velocity using the VASA-seq from E8.5 and E9.5 mouse embryo. Arrows indicate the average speed on a defined grid (number of grids=20). Blue points represent E9.5 and red points represent E8.5.
